# Supplementary material for: Burrowing Behavior as Robust Parameter for Early Humane Endpoint Determination in Murine Models for Pancreatic Cancer
Source: Animals (Basel). 2025 Apr 28;15(9):1241. doi: 10.3390/ani15091241 (PMC12071103; doi:10.3390/ani15091241)
Supplement: Supplementary file 1 [file animals-15-01241-s001.zip › Supplementary Material Table S1-5 and Fig S1_JB.pdf]

animals

MDPI

Supplemental Material

Burrowing Behavior as Robust Parameter for Early Humane Endpoint Determination in Murine Models for Pancreatic Cancer

Jakob Brandstetter [1], Lisa Hoffmann [1], Ingo Koopmann [1], Tim Schreiber [1], Benjamin Schulz [1], Stephan Patrick Rosshart [2,3], Dietmar Zechner [1], Brigitte Vollmar [1] and Simone Kumstel [1,*]

[1] Rudolf-Zenker-Institute of Experimental Surgery, University Medical Center Rostock, 18057 Rostock, Germany; jakob.brandstetter@uni-rostock.de (J.B.); lisa.hoffmann@uni-rostock.de (L.H.); ingo.koopmann@uni-rostock.de (I.K.); tim.schreiber@med.uni-rostock.de (T.S.); benjamin8989@web.de (B.S.); dietmar.zechner@uni-rostock.de (D.Z.); brigitte.vollmar@med.uni-rostock.de (B.V.)

[2] Department of Microbiome Research, University Hospital Erlangen, Friedrich-Alexander-Universität Erlangen-Nürnberg (FAU), 91054 Erlangen, Germany; stephan.rosshart@uk-erlangen.de

[3] Department of Medicine 1, University Hospital Erlangen, Friedrich-Alexander-Universität Erlangen-Nürnberg (FAU), 91054 Erlangen, Germany

* Correspondence: simone.kumstel@uni-rostock.de; Tel.: +49-381-4942512

Figure S1. Experimental design for the monitoring of welfare parameters in the different murine pancreatic cancer models

Table S1. Assignment of the mice to the specific PDA models and therapy groups

Table S2. Clinical score sheet for murine Panc02 cell induced PDA model.

Table S3. Clinical score sheet for murine 6606PDA cell induced metastasized PDA model.

Table S4. Statistics for data of figure 1, 3, 5 and 7

Table S5. Reduction of burrowing behavior (17 h) for the orthotopic PDA models and different treatment groups

 https://doi.org/10.3390/ani15091241

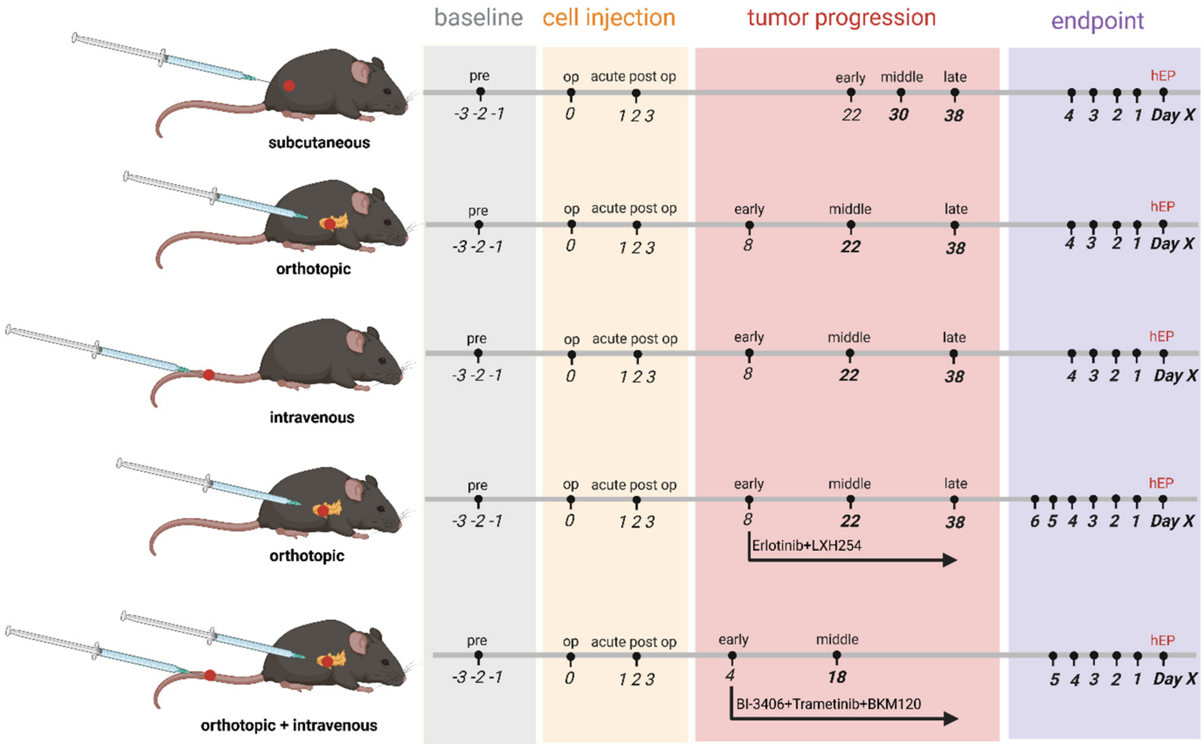

Figure S1. Time schedule of the experiment for the monitoring welfare parameters in the different murine pancreatic cancer models. The welfare parameters burrowing behavior, nesting activity, perianal temperature and mouse grimace scale were assessed before tumor cell injection (pre), after tumor cell injection (op, acute post op) and during the early, middle and late phase of tumor progression. The days of analysis during tumor progression differed between the distinct pancreatic cancer models, due to varying degrees of rapid tumor progression. As soon as criteria from the score sheets were noticed, all parameters were assessed daily until the occurrence of endpoint criteria. Created with Biorender.

Table S1. Assignment of the mice to the specific PDA models and therapy groups

animal model/ injection site	cell line	strain	treatment	gender	number of animals
subcutaneous	Panc02	C57BL/6J	no treatment	♀	5
				♂	4
intravenous	Panc02	C57BL/6J	no treatment	♀	4
				♂	5
orthotopic	Panc02	C57BL/6J	no treatment	♀	5
				♂	4
orthotopic	Panc02	C57BL/6J	Vehicle	♀	3
				♂	2
			Erlotinib	♀	4
				♂	3
			LXH254	♀	3
				♂	3
			Erlotinib + LXH254	♀	2
				♂	4
orthotopic + intravenous	6606PDA	C57BL6/NTac	Vehicle	♂	6
			Trametenib + BI-3406	♂	2
			Trametenib + BI-3406+ BKM120	♂	3
orthotopic + intravenous	6606PDA	C57BL6NTac/wild	Vehicle	♂	3
			Trametenib + BI-3406	♂	1
			Trametenib + BI-3406 + BKM120	♂	1

Table S2. Clinical score sheet for murine Panc02 cell induced PDA model.

Observations	Score
**I Body weight**	
I-a decreased > 10% (compared to initial weight)	3
I-b decreased > 15% (compared to initial weight)	4
**II General condition**	
appearance	1 (A)
II-a tooth displacement, too long teeth	2
II-b fur dull, ruffled or untended	2
II-c eyes unclear or squinted	3
II-d untended orifices of the body	3
II-e abnormal posture	3
II-f dehydration	
impairments/abnormalities	3
II-g short spasms or temporary paralysis symptoms	4
II-h persistent (>30’) cramping or paralysis	
II-i abnormal respiratory sounds or breathing, animal feels cold	4
II-j state of cachexia (body condition score: BC 2)	4
**III Spontaneous behavior**	
III-a animal is passive or overactive	2
III-b pronounced apathy, hyperkinesia, isolation	4
III-c squeaking due to pain	4
III-d self-mutilation	4
**IV Flight behavior after contact**	
IV-a animal is moderately passive or overactive	2
IV-b distinct apathy or hyperkinesia	4
**V Process-specific criteria**	
V-a wound healing disorder	2
V-b opening of the sutures by biting	1 (B)
V-c local inflammation	2
V-d ascites	4
V-e rectal bleeding/anal prolapse	4
V-f tumor size ⩾ 2000 m³	4
V-g tumor-related impairment of motion	4
V-h tumor ulceration	4
V-i bloated belly and bent body posture	4

Score points given per line as soon as one criteria applies. Even with several positive results per line, there is no addition of the points per line.

Procedures

Single score	Total score	Distress level	Measures
A		mild	Shorten teeth.
B		moderate	Inform the person in charge of the experiment. If necessary, anesthetize the animal and close the wound. Document it.
1		mild	Inform the person in charge of the experiment. A sufficient frequency of observation is necessary, consider treatment options and document it.
2-3		moderate	Inform the person in charge of the experiment. Daily observation of the animal is necessary, consider treatment options and document it.
4		severe	In agreement with the person in charge euthanasia (preferably painless after anesthesia) has to be performed. Document it.
	3-4	mild	Inform the person in charge of the experiment. Daily observation of the animal is necessary, consider treatment options and document it.
	5-10	moderate	Inform the person in charge of the experiment. Euthanasia or treating the animal plus daily observation of the animal is necessary. Document it.
	> 11	severe	In agreement with the person in charge euthanasia (preferably painless after anesthesia) has to be performed. Document it.

Published in: Schreiber, T.; Koopmann, I.; Brandstetter, J.; Talbot, S.R.; Goldstein, L.; Hoffmann, L.; Schildt, A.; Joksch, M.; Krause, B.; et al. Evidence-Based Severity Assessment of Animal Models for Pancreatic Cancer. Biomedicines 2024, 12, 1494. https://doi.org/10.3390/biomedicines12071494

Table S3. Clinical score sheet for murine 6606PDA cell induced metastasizing PDA model.

Observations	Score
**I Body weight**	
I-a decreased > 10% (compared to initial weight)	2
I-b decreased > 20% (compared to initial weight)	5
**II General condition**	
appearance	
II-a tooth displacement, too long teeth	1 (A)
II-b fur dull, ruffled or untended	2
II-c eyes unclear or squinted	2
II-d untended orifices of the body	3
II-e abnormal posture	3
II-f dehydration	3
impairments/abnormalities	
II-g short spasms or temporary paralysis symptoms	3
II-h persistent (>30') cramping or paralysis	5
II-i abnormal respiratory sounds or animal feels cold	5
**III Spontaneous behavior**	
III-a animal is passive or overactive	2
III-b pronounced apathy, hyperkinesia, isolation	4
III-c squeaking due to pain	5
III-d self-mutilation	5
**IV Flight behavior after contact**	
IV-a animal is moderately passive or overactive	2
IV-b distinct apathy or hyperkinesia	5
**V Process-specific criteria**	
V-a wound healing disorder	2
V-b opening of the sutures by biting	1 (B)
V-c local inflammation	2
V-d ascites	4

Score points given per line as soon as one criteria applies. Even with several positive results per line, there is no addition of the points per line.

Procedures

Single score	Total score	Distress level	Measures
A		mild	Anesthetize animal and shorten teeth. Document it.
B		moderate	Inform the person in charge of the experiment. If necessary, anesthetize the animal and close the wound. Document it.
1		mild	Inform the person in charge of the experiment. A sufficient frequency of observation is necessary, consider treatment options and document it.
2-4		moderate	Inform the person in charge of the experiment. Daily observation of the animal is necessary, consider treatment options and document it.
5		severe	In agreement with the person in charge euthanasia (preferably painless after anesthesia) has to be performed. Document it.
	3-4	mild	Inform the person in charge of the experiment. Daily observation of the animal is necessary, consider treatment options and document it.
	5-15	moderate	Inform the person in charge of the experiment. Euthanasia or treating the animal plus daily observation of the animal is necessary. Document it.
	> 15	severe	In agreement with the person in charge euthanasia (preferably painless after anesthesia) has to be performed. Document it.

Published in: Kumstel, S.; Tang, G.; Zhang, X.; Kerndl, H.; Vollmar, B.; Zechner, D. Grading Distress of Different Animal Models for Gastrointestinal Diseases Based on Plasma Corticosterone Kinetics. Animals 2019, 9, 145. https://doi.org/10.3390/ani9040145

Table S4. Statistics for data of figure 1, 3, 5 and 7

figure	statistic model	effects	F (DFn, DFd)	p-value
1A	Two-Way ANOVA	time	F (3.247, 6494) = 20.60	0.0367*
		model	F (2, 20) = 4.431	<0.0001*
		time x model	F (12, 120) = 1.934	0.0255*
1B	Two-Way ANOVA	time	F (2.87,56.94) = 5.792	0.0104*
		model	F (2,20)= 5.792	0.0104*
		time x model	F (12, 120) = 5.293	<0.0001*
1G	Mixed-effects analysis	time	F (2.210, 25.41) = 7.324	0.0024*
		model	F (2, 46) = 1.194	0.3122
		time x model	F (8, 46) = 2.509	0.237*
3A	Two-Way ANOVA	time	F (2.468, 49.36) = 17.65	<0.0001*
		treatment	F (3, 20) = 0.4485	<0.0001*
		time x treatment	F (21, 140) = 0.5400	0.9491
3B	Two-Way ANOVA	time	F (2.903, 58.07) = 74.80	<0.0001*
		treatment	F (3, 20) = 0.3658	0.7785
		time x treatment	F (21, 140) = 1.258	0.2142
5A	Two-Way ANOVA	time	F (1.956, 15.65) = 49.30	<0.0001*
		treatment	F (2, 8) = 0.6431	0.5508
		time x treatment	F (14, 56) = 0.7312	0.7344
5B	Two-Way ANOVA	time	F (2, 113) = 34.07	<0.0001*
		treatment	F (2, 8) = 0.7245	0.5138
		time x treatment	F (14, 56) = 1.055	0.4165
7A	Two-Way ANOVA	time	F (1.53, 3.072) = 9.335	0.0510
		treatment	F (2, 2) = 1.561	0.3905
		time x treatment	F (14, 14) = 0.772	0.6833
7B	Two-Way ANOVA	time	F (1.564, 3.128) = 40.61	0.0061*
		treatment	F (2, 2) = 0.5023	0.6657
		time x treatment	F (14, 14) = 1.939	0.1139
7D	Mixed-effects analysis	time	F (1.058, 1.693) = 5.351	0.1709
		treatment	F (1, 8) = 1.482	0.2581
		time x treatment	F (5, 8) = 0.2933	0.9037

Table S5. Reduction of burrowing behavior (17 h) for the orthotopic PDA models and different treatment groups

cell line/ strain	treatment	days before hep	animal 1	animal 2	animal 3	animal 4	animal 5	mean	reduction [%]
Panc02 B6	non	tp	105	190	200	200		173.75	
		2			4	0		2	98.85
		1	21	0	0	1		5.5	96.83
Panc02 B6	vehicle	tp	200	200	200	200		200	
		2	27	1	14	88		32.5	83.75
		1	65	0	61	67		48.25	75.88
Panc02 B6	Erlotinib	tp	195	200	200	126	200	184.20	
		2		155	17	27	13	53.0	71.23
		1	42	2	0	4	0	9.6	94.79
Panc02 B6	LXH-254	tp	200	200	200	189		197.25	
		2	123		58	37		72.67	63.16
		1	11	1	0	4		4	97.97
Pan02 B6	LXH-254 + Erlotinib	tp	200	197	200	199	200	199.20	
		2			40	103		71.50	64.11
		1	1	4	10	71	25	22.20	88.86
6606PDA B6NTac	vehicle	tp	195	197	169	136	140	167.4	
		2	18	23	165	36	32	54.80	67.26
		1	4	28	53	14	36	27.0	83.87
6606PDA B6NTac	Tram + BI	tp	200	193				196.5	
		2	184	33				108.5	44.78
		1	0	9				4.5	97.70
6606PDA B6NTac	Tram + BI + BKM	tp	170	23	148			113.67	
		2	21	46	109			58.67	48.39
		1	42	4	37			27.67	75.66
6606PDA Wildling	vehicle	tp	200	200	97			165.67	
		2		107	56			81.5	50.80
		1	15	8	100			41	75.25
